# Historical trends in breast cancer presentation among women in Pakistan from join-point regression analysis

**DOI:** 10.12669/pjms.40.1.7123

**Published:** 2024

**Authors:** Sidra Zaheer, Farah Yasmeen

**Affiliations:** 1 Sidra Zaheer, Ph.D. School of Public Health, Dow University of Health Sciences, OJHA Campus, SUPARCO Road, Gulzar e Hijri, Karachi, Pakistan, Department of Statistics, University of Karachi, Karachi, Pakistan; 2 Dr. Farah Yasmeen, Ph.D. Department of Statistics, University of Karachi, Karachi, Pakistan

**Keywords:** Annual percentage change, Breast cancer, Incidence, Join-point regression, Trend analysis

## Abstract

**Objective:**

Breast cancer prevalence in Pakistan is among the highest in Asian countries. Recent changes in the temporal trends of breast cancer are largely unknown and examination of these trends can provide a direction in national planning for future health programs. The aim of this study was to examine recent changes in breast cancer incidence in Pakistan.

**Methods:**

A total of 9,771 diagnosed breast cancer women were registered from 2004 to 2015 in three hospitals of Karachi. Join-point regression analysis was applied to assessing the age-standardized breast cancer presentation trends for each five-year age group. Age-specific average annual percentage changes were also calculated to assess the proportion of women with increased or decreased incidence of breast cancer.

**Results:**

Age-standardized rates of breast cancer steadily increased from 24.7 per 100,000 in 2004, to 45.4 per 100,000 in 2015. The analytical trend based on the join-point model showed an average percentage increase breast cancer prevalence of 5.4 (95% CI = 3.1, 7.8). Significantly higher prevalence rates were identified among women aged 40-44 years and 65-69 years with an average percentage change of 18.5 (95% CI = 2.3, 37.2) and 14.3 (95% CI = 2.2, 27.9) respectively in the four years from 2011 to 2015.

**Conclusion:**

The findings indicate the trend in age-standardized prevalence increased significantly in all age groups with noticeably larger increases observed among older and post-menopausal women from 2011 to 2015. The results warrant the need for more targeted interventions to high-risk groups and a sound foundation for cancer control program planning and policy development in Pakistan to reduce the increasing incidence of breast cancer.

## INTRODUCTION

Breast cancer is not only the most frequently diagnosed cancer but is also the leading cause of death among women. Worldwide over 2.3 million newly diagnosed cases of breast cancer are forecast, constituting 1/4 cancer cases and 1/6 cancer deaths among women in the year 2020.^1^ The National Cancer Institute report an estimated 266,120 (15.3%) new breast cancer cases and 40,920 (6.7%) deaths in 2018.^2^ According to the Asia-global cancer observatory, in 2020 there were 4.4 million women newly diagnosed cancer cases in the Asia region, with approximately 23% (1.1 million) due to breast cancer.^3^ Although breast cancer incidence is lower in Asia compared to Western countries, recent findings indicate the burden of breast cancer is also rising rapidly in Asian countries.^1^,^4^-^6^

Pakistan is the fourth most populous country in Asia and one of the top five countries containing 75% of Asia’s population. Given Pakistan’s lower-middle income status in the region, the country faces a considerable burden of cancers, with significant rising prevalence trends.^2^ Breast cancer accounted for 28.7% (25,928) of all new cases and 11.7% (13,725) of all cancer deaths in Pakistan.^1^ Currently, one in every nine women is at risk of breast cancer with the age-standardized breast cancer incidence in Pakistan being among the highest in Asia.^6^-^8^ Coupled with the higher breast cancer incidence to neighboring countries like Iran and India, breast cancer is the most common cause of cancer among women in Pakistan.^9^,^10^

The age-standardized prevalence of breast cancer among women in Pakistan has increased rapidly in the last twenty years.^6^,^7^,^11^ The age-specific incidence of breast cancer in Pakistan has also rapidly increased following menopause, and has been found to be most apparent in women over 50 years of age,^12^ with other studies confirming that the peak age of breast cancer prevalence in Pakistan was between 45-59 years.^6^,^11^,^13^

However, previous studies have only focused on descriptive results and did not apply any statistical modeling to report time trends of breast cancer prevalence according to age groups in Pakistan. Identification of age-specific prevalence trends and changing patterns over time can play an important role in informing policymakers and health officials to better plan and prioritize disease control activities, including improved treatments, and evaluation of individual patient care.

The aim of this study was to evaluate trends in the incidence rate of breast cancer and patterns of cancer occurrence by age group in Pakistan from 2004 to 2015 applying join-point regression analysis to identify specific risk profiles of breast cancer patients. To the best of our knowledge, this is the first study on trend analysis of breast cancer prevalence conducted in Pakistan using join-point regression analysis and reporting average annual percentage changes at a national level.

## METHODS

### Data source

The data used for this study were collected from the three hospitals in Karachi, the largest city in Pakistan with an estimated population of over 16 million^14^, with the most accessible health facilities for patients from Karachi, Sindh Province and other regions in Pakistan. Hospitals for the study included Jinnah Post Graduate Medical Centre (JPMC), Karachi Institute of Radiotherapy and Nuclear Medicine (KIRAN), and Civil Hospital, Karachi (CHK) with these institutions being the leading cancer and regional referral centers serving as collection units of breast cancer cases in the Sindh Province.^12^

### Study population

Available medical records were sources of breast cancer patients from the three hospitals who were diagnosed between 2004 and 2015. A total of 9771 diagnosed cases of registered breast cancer patients were included. The primary medical reports of all qualified patients were collected and general information, risk factors, diagnostic tests were recorded. The registered cases with missing information such as age and duplicate information (n=197) were excluded from the study to eliminate any confounding effect. The international classification of diseases-oncology was used for patient diagnosis. Diagnosis of breast cancer based on histopathology and radiology reports (biopsy/CT/MRI reports) confirmed by concerned doctors were recorded on first visit of the patients. Hospital selection, case sampling, and data collection methods have been previously described in detail.^12^

### Observed and Age-standardized incidence rates (ASIR)

After collection, breast cancer incidence cases were sorted into age-specific categories. We used standardization of age as 15-19, 20-24, 25-29, 30-34, 35-39, 40-44, 45-49, 50-54, 55-59, 60-64, 65-69, 70-74, 75+ years. To express the age-specific breast cancer incidence rates per 100,000 annually, we divided the number of new cases of breast cancer by the corresponding annual population at risk.^12^ ASIRs were also reported to standardize the effect of age over time. The direct standardization method was used for adjusting the age effect using the WHO World Standard Population.^15^

### Join-point regression analysis (JRA)

The trends in age-standardized incidence rates were explored by applying Join-point Regression Analysis (JRA). JRA identifies statistically significant calendar years (Join-points) and gives the annual percent change (APC) in each trend segment using a Monte Carlo permutation method. This technique also provides an outcome with the log-linear regression model of best fit. This approach is widely used to estimate the trend of incidence or mortality rates in several diseases.^16^-^18^ The purpose of that approach is to identify possible join-points where a significant change in a trend occurs. Statistical analysis was performed using the Join-point Regression Program, Version 4.9.0.1^19^ and significance was taken as p-value<0.05.

### Ethical approval

Ethics approval for the study was provided by the Pakistan Atomic Energy Commission of the KIRAN hospital, Karachi, Pakistan. (Reference Number: KIRAN-Estt.2(67)/14). Formal written approval from hospital administration was obtained regarding the use of anonymized and de-identified medical records. Consent to participate was not applicable to this study which was a secondary analysis of de-identified retrospective medical records.

## RESULTS

### Observed breast cancer prevalence rates

The increasing trend of the age-specific incidence rates, which is more evident in women aged 35 years and above during the period 2004-2015. The highest breast cancer prevalence rates were observed in women aged 60–64 years while there was a sharp increase in incidence between 2012 and 2015. The trend was considerably lower and stable in ages 15 to 34 years, while the prevalence rates for women aged 35 years and older increased overall with slight variations.

During the study period, ASIR steadily increased from 24.7 per 100,000 in 2004 to 45.4 per 100,000 in 2015 (Table-I). Similarly, the analytical trend based on join-point analysis showed an increased breast cancer incidence trend by an average annual percentage change (APC) of 5.4 (95% CI = 3.1, 7.8). Join-point regression identified no significant cut-point (0 join-point), indicating that the increasing trend in incidence remained steady from 2004 to 2015 (Fig.2).

**Table-I:** Breast cancer incidence, crude incidence rate and age-standardized incidence rates (per 100,000) between 2004 to 2015.

Years	Incidence	CIR^A^	ASIR^B^
2004	573	17.4	24.7
2005	619	18.2	26.3
2006	611	17.4	25.8
2007	641	17.7	27.0
2008	850	22.8	34.1
2009	616	16.0	25.1
2010	696	17.6	27.4
2011	725	17.8	28.0
2012	826	19.6	31.0
2013	1119	25.8	39.2
2014	1186	26.5	41.1
2015	1309	28.3	45.4

A. CIR: crude incidence rate (per 100,000 persons), B. ASIR: Age-standardized incidence rate (per 100,000 persons).

**Fig.1:**
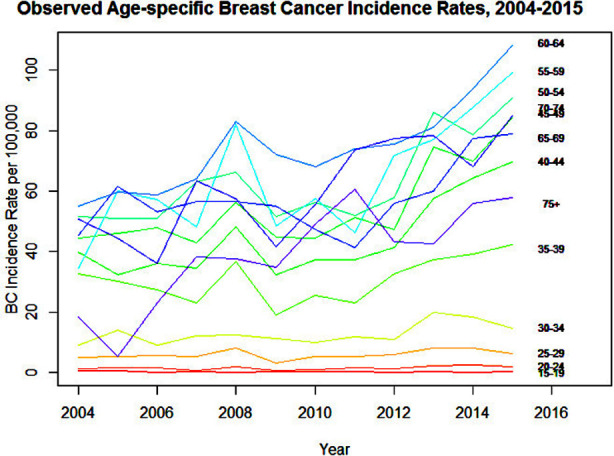
Observed breast cancer incidence rates (per 100,000 women) by age-group from 2004-2015.

**Fig.2:**
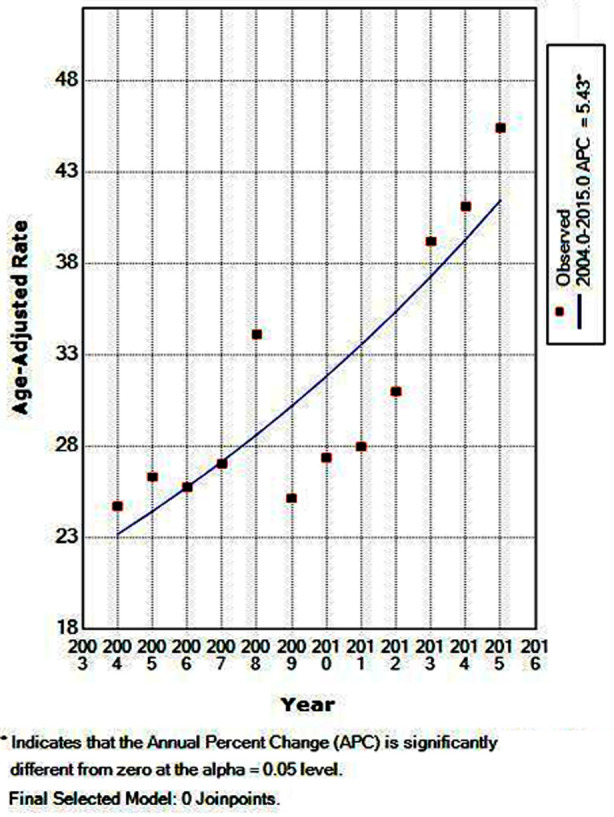
Trends in estimated age-standardized breast cancer incidence rates (per 100,000 women) from 2004-2015. ^APC (Annual percentage change) represents significant difference from zero at alpha=0.05.

### Age-specific breast cancer prevalence trends

**Fig.3:**
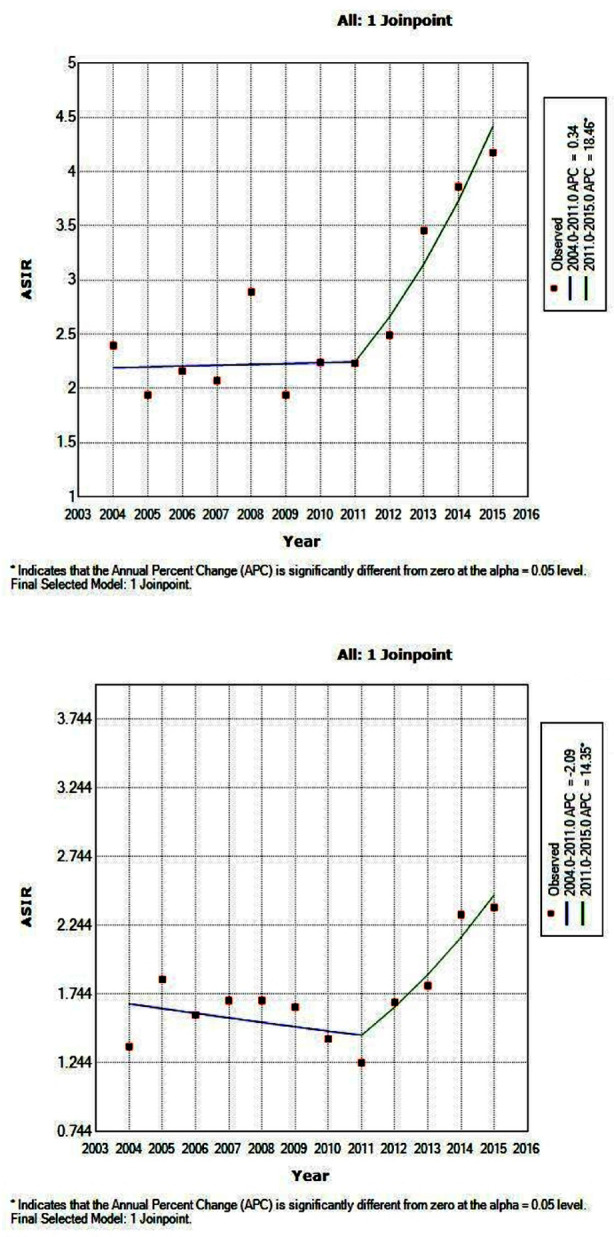
Trends in estimated age-standardized breast cancer incidence rates (per 100,000 women) for age group 40-44 (above image) and for age group 65-69 (lower image). ^APC (Annual percentage change) represents significant difference from zero at alpha=0.05.

When age-specific trends were evaluated, the APCs indicate the trend in age-standardized breast cancer incidence increased significantly in all age groups over years, with noticeable increases observed for the women aged 40 years and above. A significant join-point was noted in the 2011 providing two distinct trends from 2004-2011 and from 2011 to 2015. The prevalence rates significantly increased among women aged 40-44 years and 65-69 years with an APC of 18.5 (95% CI = 2.3, 37.2) and 14.3 (95% CI = 2.2, 27.9) respectively from 2011 to 2015 (Table-II; Fig.3).

**Table-II:** Age-specific breast cancer incidence rates for each trend among women in Karachi.

	Full Period (2004-2015)		Trend 1 (2004-2011)		Trend 2 (2011-2015)	

Age-group	aAPC^C^	95% CI	APC	95% CI	APC	95% CI
15 – 19*	-9.3	-17.4, 0.3				
20 – 24*	3.5	-3.8, 11.3				
25 – 29*	3.0	-1.7, 7.9				
30 – 34*	4.5^	0.5, 8.6				
35 – 39*	2.8	-1.6, 7.5				
40 – 44**	6.6^	0.9, 12.6	0.3	-5.7, 6.8	18.5^	2.3, 37.2
45 – 49*	5.0^	2.0, 8.2				
50 – 54*	4.5^	1.7, 7.5				
55 – 59*	6.3^	1.9, 10.9				
60 – 64*	5.1^	3.3, 6.9				
65 – 69**	3.6	-0.7, 8.0	-2.1	-6.6, 2.7	14.3^	2.2, 27.9
70 – 74*	6.1^	2.6, 9.7				
75+*	15.2^	5.4, 26.0				

C. ^APC or aAPC represents significant difference from zero at alpha=0.05, aAPC: average annual percentage change,

* Final selected model 0 Join-point;

** Final selected model 1 Join-point.

## DISCUSSION

This study presents an application of join-point regression analysis to identify the time trends of breast cancer prevalence rates. The analysis provides greater understanding of join-points in different years, to find when breast cancer incidence started rising, reached its peak, at what speed and type of changes occurred of age and time-related differences in the incidence rate of breast cancer among women of Karachi, Pakistan.

The current study showed a significant increase in breast cancer presentation trend with an average APC of 5.4. The prevalence rates of all the age groups have continued to increase since 2000,^7^ and the same trend has been observed for the females in other local studies conducted in Pakistan.^6^,^13^ During the study period, ASIR steadily increased from 24.7 per 100,000 in 2004 to 45.4 per 100,000 in 2015, this increasing trend was similar to the reported findings from other developing and developed countries.^5^,^20^,^21^

Surprisingly, the highest presentation trend was observed in age groups of 40-44 years and 65-69 years in the country with an average APC of 18.5 and 14.3 respectively. The overall breast cancer prevalence occurred more rapidly among older and post-menopausal women, this finding may reflect the high use of menopausal hormone therapy in older ages and the lack of awareness about early screening methods in Pakistani women.^22^-^24^ Moreover, poor socioeconomic status, illiteracy and lack of access to healthcare facility may have contributed to more cases of breast cancer being diagnosed in the older ages.^25^,^26^

The results infer an increase in the prevalence rate of elderly breast cancer coherent to the trends observed among women in other studies conducted worldwide.^20^,^21^,^27^,^28^ This suggests that increases in the adoption of westernized lifestyle, poor dietary habits, physical inactivity and obesity are leading risk factors for increasing breast cancer incidence in the region.^4^,^29^ However, in the United States, breast cancer incidence rates showed a significant decline in all five-year age groups from above 45 years or older. Studies suggested that the decrease in prevalence may explain the early benefit of the reduced use of hormone replacement therapy and the saturation in screening mammography.^30^,^31^

We observed higher decreasing incidence rates for younger ages 15 to 19 years, which imitate a lack of awareness and absence of mammographic screening programs targeting younger women in Pakistan.^23^,^24^ In the present study, a significant increasing trend in incidence was noted after 2011 making two distinct trends from 2004-2011 and from 2011 to 2015. The definite reasons for the relatively sharp increasing trends in our setting were uncertain; however, the present study suggests that largely increase in screenings and registrations as well as advances in data management may be part of the explanation.^20^,^32^

### Strengths of the study

This study has a number of important strengths. First, we applied the join-point regression approach to estimate the temporal trends of age-specific incidence of breast cancer in different years. Join point models developed to uncover changes in time trend of mortality and incidence of disease, as well as join-point regression analysis is suitable for several fields of the epidemiologic investigations to identify the temporal trends of several measures^18^,^33^,^34^ Moreover, for the analysis of trends using join-point models, “Join-point Regression Program” software was used. This program developed by National Cancer Institute (NCI) is a user-friendly software and is available on the website for public use free of charge.^19^

### Limitations of the study

There is a possibility of loss of information for those patients who had no access to any health care facility or those who were diagnosed at other institutes or hospitals. Although the data used in this study was collected from three major centers, which can provide vital clinical information on breast cancer prevalence and allow accurate estimates of national patterns when there is no screening or registration program at national level in the country.^12^,^13^ In spite of some limitations, our study is an essential and novel study countrywide. This study presents the application of advanced join point regression models and investigation of presentation trends in breast cancer at national level.

## CONCLUSION

The findings indicate the trend in age-standardized presentation increased significantly in all age groups with noticeably larger increases observed among older and post-menopausal women from 2011 to 2015. The results warrant the need for more targeted interventions to high-risk groups and a sound foundation for cancer control program planning and policy development in Pakistan to reduce the increasing incidence of breast cancer.

### Authors’ contribution

**SZ** conceived the idea of the work and statistical analysis.

**SZ and FY** were involved in interpretation of the results and writing of the manuscript.

Both authors read and approved the final manuscript.
